# Supplementary material for: Vectors as Sentinels: Rising Temperatures Increase the Risk of Xylella fastidiosa Outbreaks
Source: Biology (Basel). 2022 Aug 31;11(9):1299. doi: 10.3390/biology11091299 (PMC9495951; doi:10.3390/biology11091299)
Supplement: Supplementary file 1 [file biology-11-01299-s001.zip › SI_methods_Tables_Figures.pdf]

Supplementary materials to

Vectors as sentinels: Rising temperatures increase the risk of *Xylella fastidiosa* outbreaks.

Pauline Farigoule[1,2], Marguerite Chartois[1,3], Xavier Mesmin[1,3], Maxime Lambert[1], Jean-Pierre Rossi[1£], Jean-Yves Rasplus[1£] and Astrid Cruaud[1£]

[1] CBGP, INRAE, CIRAD, IRD, Institut Agro, Univ Montpellier, 34988 Montferrier-sur-Lez, France; [2] AgroParisTech, 91120 Palaiseau, France; [3] AGAP Institut, INRAE, CIRAD, Institut Agro, Univ Montpellier, 20230 San Giuliano, France; [£]These authors are joint senior authors on this work.

Table of contents

Supplementary methods 2

Molecular approach 2

Analysis of sequence data 3

Climate profile of the sampling sites 4

Generalized Linear Mixed Models 5

Projections of climate data for variables with significant effect on prevalence 5

Species distribution modeling 6

Table S1 9

Table S2 9

Table S3 10

Table S4 11

Table S5 12

Table S6 13

Figure S1 14

Figure S2 15

Figure S3 16

Figure S4 17

Figure S5 18

Cited references 19

Supplementary methods

Molecular approach

Quantitative and conventional PCR are the established gold standard methods to test the presence of *Xf* in plants [1] but drawbacks regarding sensitivity and versatility have been highlighted for vectors [2]. In a previous study, we relied on nested PCR and Sanger sequencing to assess prevalence in populations of vectors. Here we developed a high-throughput approach based on two PCR steps followed by MiSeq sequencing of one of the housekeeping genes of the multi-locus sequence typing scheme of *Xf* [3], *leuA*. In the first PCR, the target gene region is amplified using specific primers flanked by tails. These tails allow for a second PCR reaction to add Illumina adapters and indexes to multiplex samples.

DNA was extracted from single specimens following Cruaud et al. [2] to reduce the impact of PCR inhibitors. The 2 step PCR protocol was adapted from Cruaud et al. [4]. Specific primers for PCR1 were designed from an alignment of all *leuA* alleles available in pubMLST (http://pubmlst.org/xfastidiosa/) [last access may 2021; no new *leuA* allele was added when this manuscript was submitted in may 2022] using Geneious Prime 2020.0.4 (https://www.geneious.com) and PerlPrimer [5]. The target fragment (462-472 bp to obtain two overlapping fragments with a MiSeq Reagent kit 2x250 bp when the target+adapters construction is sequenced) allows discrimination between the three main subspecies of *Xf* (*Xf fastidiosa*, *Xf multiplex* and *Xf pauca*) [6]. Four PCR replicates were performed per DNA insect to reduce false negatives. Primers, mix and cycling parameters used in PCR1 are provided in Table S4. During PCR2, a unique combination of 9-nt indexes was assigned to each specimen to track index hopping (same combination of indexes for all four replicates). Indexes were taken from Martin [7]. Primers, mix and cycling parameters used in PCR1 are provided in Table S5. Two negative controls (DNA extraction and PCR) were used on each plate. Amplification was revealed by agarose gel electrophoresis (1% agarose gel stained after electrophoresis using ethidium bromide). When at least one replicate was positive, 1 µL of all four PCR2 products was included in the final pool. PCR negative controls (n=3) were included to the final pool. The final pool was purified on gel (1.25% low-melting agarose gel) with a PCR clean-up and gel extraction kit (Macherey-Nagel, Germany; final elution volume = 40 µL of NE buffer). Purified DNA was quantified with an Agilent Bioanalyzer (DNA 1000 LabChip kit (Agilent Technologies, USA)) and qPCR (Library Quantification Kit—Illumina/Universal from KAPA (KK4824)). 200 mM Tris-HCl pH7 was added to the mix to neutralize NaOH added in the

denaturation step before loading the library on the MiSeq flowcell. Sequencing was performed on a MiSeq system using a MiSeq Reagent Kit v2 (500 cycles) with a PhiX control spike in of 5%. Image analysis, base calling and data quality assessment were performed on the MiSeq instrument. Artificial combinations of indexes (n=3) were introduced in the sample sheet to quantify index hopping.

Instead of using purified DNA from inactivated bacteria as positive control and to optimize PCR conditions we used a synthetic target. This target was designed from the *leuA_2* allele. Three mutations were introduced at both ends to create a variant that was unlikely to be observed *in natura* to make detection of potential cross-contamination more straightforward. Positive controls contained 1000 copies of the synthetic target mixed with 3 µL of Nuclease-Free Water (Qiagen).

Synthetic target (based on leuA_2 with mutations highlighted in red):
5'GGTGCACGCCAAATCGAATGT**T**CT**T**TC**T**ATGGCATTGGTGAGCGCGCTGGTAACTGCGCGCTGGAAGAACTCACTATGGTG
TTGAAAGTACGCAACGCGTTTTACAACATTGATACTTCGATCCACACATCACGTATCGTCTCCACCTCCCAGTTACTGCAACG
ATTGGTTGGCATGCCCGTGCAACGTAACAAGGCAGTAGTAGGTGCCAATGCCTTTGCACATGAATCGGGTATCCATCAGCAC
GGTATGCTGCGCCATCGCGGCACCTACGAAATCATGCGTCCACAAGAAGTCGGTTGGGTATGTTCGCACATGGTACTCGGCC
GCCATAGCGGCCGTGCTGCGGTCGAACAGCGTCTACGCGCACTGGGCTACTTGCTGGAGGAAGAAGATCTAAAACTGGTATT
TGAAGAATTCAA**T**CA**C**CT**A**TGTGAGAAACAGCGTTTGGTCACCGATGTCGACCT-3'

Analysis of sequence data

Analysis of raw data was adapted from Cruaud et al. [4]. Adapter trimming and selection of good quality paired reads was performed with Trimmomatic [8] (LEADING:20 TRAILING:20 SLIDINGWINDOW:4:20 MINLEN:200). Paired reads were merged with FLASH [9]) (-x 0 -m 10 -M 300). Only sequences that started and ended with the primers used for PCR1 were kept for downstream analysis (custom script). Primers were trimmed (custom script) and clustering of sequences was performed with SWARM (d=1) [10] after dereplication with VSEARCH [11]. Only clusters with more than 10 sequences were retained for downstream analysis (custom script). Potential chimeras were removed with VSEARCH (--uchime_denovo). Consensuses were aligned to the set of reference sequences available in pubMLST (http://pubmlst.org/xfastidiosa/) with MAFFT [12]. Alignment was visually inspected in Geneious R11.1.4 (https://www.geneious.com) to discard non-target amplifications. The complete analytical workflow with examples is available from https://github.com/acruaud/prevalenceXfinsectclimate_2022

Climate profile of the sampling sites

Climate variables (temperature/precipitation) were chosen considering the phenology of *P. spumarius* in Corsica [13]; literature on multiplication of *Xf* in plants and in *P. spumarius* [14–20] and, in absence of knowledge on epidemiological dynamics of *Xf multiplex* in Europe, annual fluctuations of Pierce's disease incidence in California [20,21].

Time slices - To roughly capture intra-annual variation in temperature and precipitation, years were first split into growing/feeding (March-November) and dormant (December-February) seasons for plants/insects. Growing season was further divided into three periods of interest regarding *P. spumarius* phenology in Corsica [13]: March-June (larvae and tenerals), July-August (aestivation) and September-October (adults; specimens were sampled in late October). Five time slices were thus defined.

Raw climate data - Raw data were retrieved from the SAFRAN model (Météo France), which interpolates temperature/precipitation measures made several times a day by a network of over 1,000 meteorological stations spread over the French territory [22]. SAFRAN provides daily temperature (2 m above ground) and precipitation data simulated at a resolution of 8 kilometers on an extended Lambert-II projection that were used to compute the studied climate variables.

Temperature-related variables - For each time slice, we computed the daily mean temperature (5 variables). For each time slice but the dormant season, we also computed the maximum temperature of the time slice (4 variables) and the average daily maximum temperature over the time slice (4 variables). For the dormant season, we computed the minimum temperature of the time slice (1 variable) and the average daily minimum temperature over the time slice (1 variable). Average of daily maximum /minimum temperatures were computed to smooth the impact of extreme and brief climatic events and give instead a better view of prolonged thermic stresses. Finally, for the growing season we computed the number of days with daily maximal temperature strictly greater than 16°C; 18°C; 20°C; 22°C; 24°C and 30°C (6 variables) while the number of days with daily minimum temperature strictly lower than 0°C; 2°C; 4°C and 6°C was computed for the dormant season (4 variables).

Precipitation-related variables - For each time slice but the dormant season, we computed the sum of daily precipitations (4 variables). We also computed the sum of daily precipitations for the growing season of the year Y-1 (1 variable). Indeed, Pierce's disease incidence in California seems higher following high rainfall years [21].

Thus, 25 temperature-related and 5 precipitation-related variables were computed to describe the climate profile of the sampling sites (Supplementary Table S1a).

Generalized Linear Mixed Models

GLMMs were built with the R [23] package glmmTMB [24]. Independent climate variables were built with two methods. First, a PCA was performed on the 30 climate variables (R package ade4 [25]) and scores of sampling sites on PC1 and PC2 were used as input for GLMM1. Second, a PLSR [26] was conducted to rank climate variables in decreasing importance (using the variable importance on projection, VIP [27]) regarding the correlation with *Xf* prevalence. Climate variables were selected step by step, in decreasing VIP order, with two conditions: VIP > 1 and Spearman correlation coefficient with variables selected in previous steps lower than 0.7 [28]. The climate data table for sampling sites reduced to the selected variables was used as input for GLMM2. To account for repeated measures, we added the random effect of sample site identifier [29]. Year was included as an experimental design fixed effect owing to the number of factor levels being below 5 [30]. GLMM validity (correct distribution, dispersion, frequency of outliers and homoscedasticity) was checked with the package DHARMa [31]. We tested the significance of fixed variables with type II analyses of deviance (two-sided type II Wald chi-square tests) with the R package car [32] and post-hoc pairwise comparisons of factor levels were performed with the R packages emmeans [33] and multcomp [34] (Tukey method for adjustment of p-values).

Projections of climate data for variables with significant effect on prevalence

To explore past and future climate, we relied on the CHELSA v2.1 database [35] (https://chelsa-climate.org). We selected the bioclimatic variable that was the closest to d6C_dec_fev (number of days from December to February with minimal temperature < 6°C) which was identified as the most explanatory variable of prevalence in GLMM2 (see main text results): bio11, the mean temperature of coldest quarter [36]. Spearman correlations between bio11 computed from SAFRAN data, and the 30 previously mentioned climate variables were computed to confirm that bio11 was a good proxy of d6C_dec_fev (Table S1a, Fig.S2).

Past and current climate - To obtain a data set of past climate conditions we computed bio11 for the following periods : 1901-1910, 1911-1920, 1921-1940, 1941-1960, 1961-1980, 1981-1990 and 1991-2000 with the R package dismo [37]. We used the bioclimatic variable calculated on the period 2000-2016 as a baseline representing "current" conditions, i.e. conditions experienced during the study.

Future climate - CHELSA also provides future climate projection of bioclimatic variables according to predictions of the fifth Assessment Report (AR5) of the Intergovernmental Panel

on Climate Change (IPCC) [38,39]. For 3 periods 2011-2040, 2041-2070 and 2071-2100, we considered 5 global circulation models (GCMs) : GFDL-ESM4, IPSL-CM6A-LR, MPI-ESM1-2-HR, MRI-ESM2-0, and UKESM1-0-LL) and 3 shared socioeconomic pathways (SSPs): SSP126, SSP370 and SSP585 to account respectively for the uncertainties related the evolution of physical processes in the atmosphere, oceans, cryosphere and land surface [40] and with the response of human societies to climate change [41] (see Table S1c for details).

Species distribution modeling

Occurrence data sets - For *P. spumarius*, we used occurrences from Cruaud et al. [2] supplemented by data available from GBIF (https://doi.org/10.15468/dl.2tdbuq). For *X. fastidiosa,* we considered only occurrences of *X. fastidiosa* ssp. *multiplex (Xfm)*, that was shown to occur in Corsica and identified in the insects screened in this study. Occurrence data were taken from Godefroid et al. [42] , Falsini et al. [43] (Italy, Tuscany), the European project Xfactors (https://www.xfactorsproject.eu/press_review/first-identification-of-xylella-fastidiosa-in-portugal/) (Portugal) and the French authorities (https://draaf.occitanie.agriculture.gouv.fr/xylella-fastidiosa-point-de-situation-dans-l-aude-mise-a-jour-de-l-arrete-de-a6149.html) (France).

We removed duplicate occurrence points, and points with missing geographical coordinates and obtained 10,325 and 421 valid occurrences for *P. spumarius* and *Xfm* respectively. For *P. spumarius*, the model was based on the occurrence points located in Europe (longitude: -15, +40 ; latitude +34, +53). We used the geographical thinning procedure (with a threshold of 15 km [44]) available in the R package spThin [45] to remove the sampling bias in the *P. spumarius* data set.

Climate reference data - We used the bioclim variables available from the CHELSA database for the period ranging from 1981 to 2010 [35]. These data were assumed to accurately describe the historical conditions and were used to calibrate our models. We used the climate descriptors conveying the possible constraints of temperature and precipitation (Table S3). Annual means were discarded because they do reflect average conditions rather than extreme values, which are actual ecological constrains.

Future climate data - We used the same periods, GCMs and SSPs as for "Projections of climate data for variables with significant effect on prevalence" (see above and Table S1c for details). The climate raster data sets used in this study are 1 x 1 km resolution.

Modeling framework - We modeled the geographical distribution of *P. spumarius* and *Xfm* by means of the Maxent algorithm [46]. Maxent is widely used and does not require true absence

data which is an advantage when dealing with newly introduced species in expansion for which obtaining true absence data is problematic [47].

Climate descriptors are often strongly correlated, which is known to cause problem during model calibration [48]. We used the R package usdm [48] to detect collinearity by means of the variance inflation factor (VIF). Climate variables showing VIF> 10 were discarded.

For both species, the remaining climate variables were used to perform environmental filtering to remove points carrying redundant environmental information which is known to improve species distribution models [49]. We performed a Principal Component Analysis (PCA) on the climate variables x occurrence points and divided the range of the occurrence scores upon the first component in 100 bins. The range of the second PCA axis was divided in bins of similar amplitude leading to a square grid. The thinning consisted in randomly selected one point when several points fell into a given grid cell.

We randomly generated 10 000 background data for each species [50]. For *Xfm*, background points were generated within an area containing the North American occurrences of the bacterium (minimum longitude -13, maximum longitude -70, minimum latitude 20, maximum latitude 70). *P. spumarius* has a large geographic distribution and available occurrences are much more numerous in northern Europe leading to a large-scale sampling bias that can hardly be corrected by usual correction methods. It should also be noted that signatures of positive selection associated with environmental variables have been identified suggesting that this large-range species may exhibit contrasted response to climate constraints [51]. For these reasons, we restricted the study area of *P. spumarius* to western Europe (longitude -15, 40 ; latitude 34, 53) to avoid under estimation of climate suitability of Mediterranean regions in our model. The background points were generated within this area and the model was calibrated using the occurrences located in that region.

The models were calibrated using the R package MIAmaxent [52]. MIAmaxent implements a forward stepwise selection procedure allowing to select a subset of explanatory variables during the model calibration leading to less complex models with better generalizability [53].

Model evaluation. We evaluated the model performance using the Area Under the Curve (AUC) [54] and the Continuous Boyce Index (CBI) [55]. AUC is commonly used in ecological modeling but in our situation where proper absence data are unavailable, background points are treated as absence data which is problematic [56]. We thus provide AUC for comparative purpose only. The CBI quantifies the frequency of presence points falling into different classes of climate suitability predicted by the model with their expectation under the null hypothesis of random distribution [example in 57]. It varies from +1 for perfect prediction to 0 (randomness)

and -1 for counter-prediction. The CBI was computed using the R package ecospat [58]. The CBI was computed within the areas used to generate the background points. Since model evaluation must involve points that are not used to calibrate the model, we used the occurrences that were discarded during both geographical and environmental filtering. Again, a geographical filtering procedure (threshold 25 km) was applied to correct for sampling bias of the points to be used in model evaluation.

For both Ps and Xf, we computed the highest climate suitability value at which there is no omission i.e. all the observed occurrences are located in an area predicted as suitable. The computation was based on the available occurrences located in Corsica and the climate suitability for the reference period (1981-2010). For each species, the threshold was applied to recode the consensus models corresponding to the different time periods and the different SSP considered in the study as well as for the reference period (1981-2010). Pixels associated to a climate suitability lower than the threshold were recoded as 0, and 1 otherwise. At a given period and for a given SSP, pixels associated to a value of 1 for both species indicated suitable conditions hence potential overlap. The surface of overlap was expressed in km^2.

Table S1 Raw data collected and analyzed in this study

Downloadable as an xlsx document in online Supplementary Materials.

Table S2 Results of the GLMMs

For GLMM1, scores of sampling sites on PC1 and PC2 of the PCA on the 30 climate variables were used as input variables. For GLMM2, the top explanatory variables according to the PLSR were used as input variables. d6C_dec_feb: number of days in the dormant season (December to February) with daily minimal temperature < 6°C; d30C_march_nov: number of days in the growing season (March to November) with daily maximal temperature > 30°C; prec_march_nov_n_1: sum of daily precipitations for the growing season of the year Y-1; prec_march_nov: sum of daily precipitations in the growing season (March to November); prec_july_august: sum of daily precipitations in July and August. Not significant explanatory variables are figured with a dash. df=degree of freedom.

Explanatory variables	Chi-square	df	P-value
**GLMM1 (marginal R^2 = 0.179)**			
year	12.9	3	0.005
PC1	6.7	1	0.010
PC2	-	-	-
**GLMM2 (marginal R^2 = 0.103)**			
year	13.9	3	0.003
d6C_dec_feb	6.1	1	0.014
d30C_march_nov	-	-	-
prec_march_nov	-	-	-
prec_july_august	-	-	-
prec_march_nov_n_1	-	-	-

Table S3 Climate descriptors used in species distribution modeling

bio5 = maximum temperature of warmest month
bio6 = minimum temperature of coldest month
bio8 = mean temperature of wettest quarter
bio9 = mean temperature of driest quarter
bio10 = mean temperature of warmest quarter
bio11 = mean temperature of coldest quarter
bio13 = precipitation of wettest month
bio14 = precipitation of driest month
bio16 = precipitation of wettest quarter
bio17 = precipitation of driest quarter
bio18 = precipitation of warmest quarter
bio19 = precipitation of coldest quarter

Table S4 Primers, mix and cycling parameters used in the first PCR

a. PCR1 primers

Specific primers are shown in green, tails (Illumina sequencing primers + primer for PCR2) are shown in orange. A cocktail of two forward or two reverse primers was used. Heterogeneity spacers (purple) were included in one of the two primers to increase diversity during the first sequencing cycles [59].

LeuA_F_v0	5'-TCGTCGGCAGCGTCAGATGTGTATAAGAGACAGGTGCACGCCAAATCGAATGT-3'
LeuA_F_v1	5'-TCGTCGGCAGCGTCAGATGTGTATAAGAGACAGACAGTGTGCACGCCAAATCGAATGT-3'
LeuA_R_v0	5'-GTCTCGTGGGCTCGGAGATGTGTATAAGAGACAGGGTCGACATCGGTGACCAAA-3'
LeuA_R_v1	5'-GTCTCGTGGGCTCGGAGATGTGTATAAGAGACAGCAAGAGGTCGACATCGGTGACCAAA-3'

b. PCR1 master mix

	10µL reaction
DNA extracted from one specimen of *P. spumarius*	3µL
QIAGEN Multiplex PCR Master Mix (Qiagen, Germany)	5µL
LeuA_F_v0 (10µM)	0.5µL
LeuA_F_v1 (10µM)	0.5µL
LeuA_R_v0 (10µM)	0.5µL
LeuA_R_v1 (10µM)	0.5µL

c. PCR1 cycling parameters

	**Temperature**	**Duration**	**Cycles**
Initial denaturation	95°C	15 minutes	1
Denaturation	94°C	30 seconds	
Annealing	60°C	90 seconds	35
Extension	72°C	90 seconds	
Final extension	72°C	10 minutes	1
Hold	10°C	∞	1

Table S5 Primers, mix and cycling parameters used in the second PCR

a. PCR2 primers

MiSeq adapters P5 or P7 are shown in red, linker (hybridization to first PCR products) are shown in orange. Unique combination of 9-nt indexes taken from Martin [7] were used to tag samples (blue).

forward	5'-AATGATACGGCGACCACCGAGATCTACACindexi5TCGTCGGCAGCGTC-3'
reverse	5'-CAAGCAGAAGACGGCATACGAGATindexi7GTCTCGTGGGCTCGG-3'

b. PCR2 master mix

	11µL reaction
Product of PCR1	2µL
QIAGEN Multiplex PCR Master Mix (Qiagen, Germany)	5µL
Primer i5 (3,5µM)	2µL
Primer i7 (3,5µM)	2µL

c. PCR2 cycling parameters

Step	Temperature	Duration	Cycles
Initial denaturation	95°C	15 minutes	1
Denaturation	95°C	40 seconds	
Annealing	55°C	45 seconds	10
Extension	72°C	60 seconds	
Final extension	72°C	10 minutes	1
Hold	10°C	∞	1

Table S6 Spatial overlap between *P. spumarius* (Ps) and *X. fastidiosa* (Xf) in Corsica

Overlaps were computed for the periods 2011-2040, 2041-2070 and 2071-2100 for the shared socio-economic pathway SSP126, SSP370 an SSP585. The spatial overlap for the period 1981-2010 (reference period) is 7 252 km^2. The surface of Corsica is 8 680 km^2.

period	SSP126	SSP370	SSP585
2011-2040	7538	7476	7212
2041-2070	7097	6588	5681
2071-2100	7264	3637	2518

Figure S1 Principal component analysis of the 30 climate variables used to describe the climate of the sampling sites during the 4 years of the survey

The first (PC1) and second (PC2) axes of the PCA respectively supported 56.7% and 12.9% of the climate variability. PC1 opposed plots with high temperatures to plots with low temperatures. PC2 opposed plots with high maximal temperatures and high precipitations during the previous year to plots with high precipitations. The orange arrow is the projection of prevalence as an illustrative variable. The list of climate variables is available from Table S1a. Figure made with the R [23] package factoextra [60].

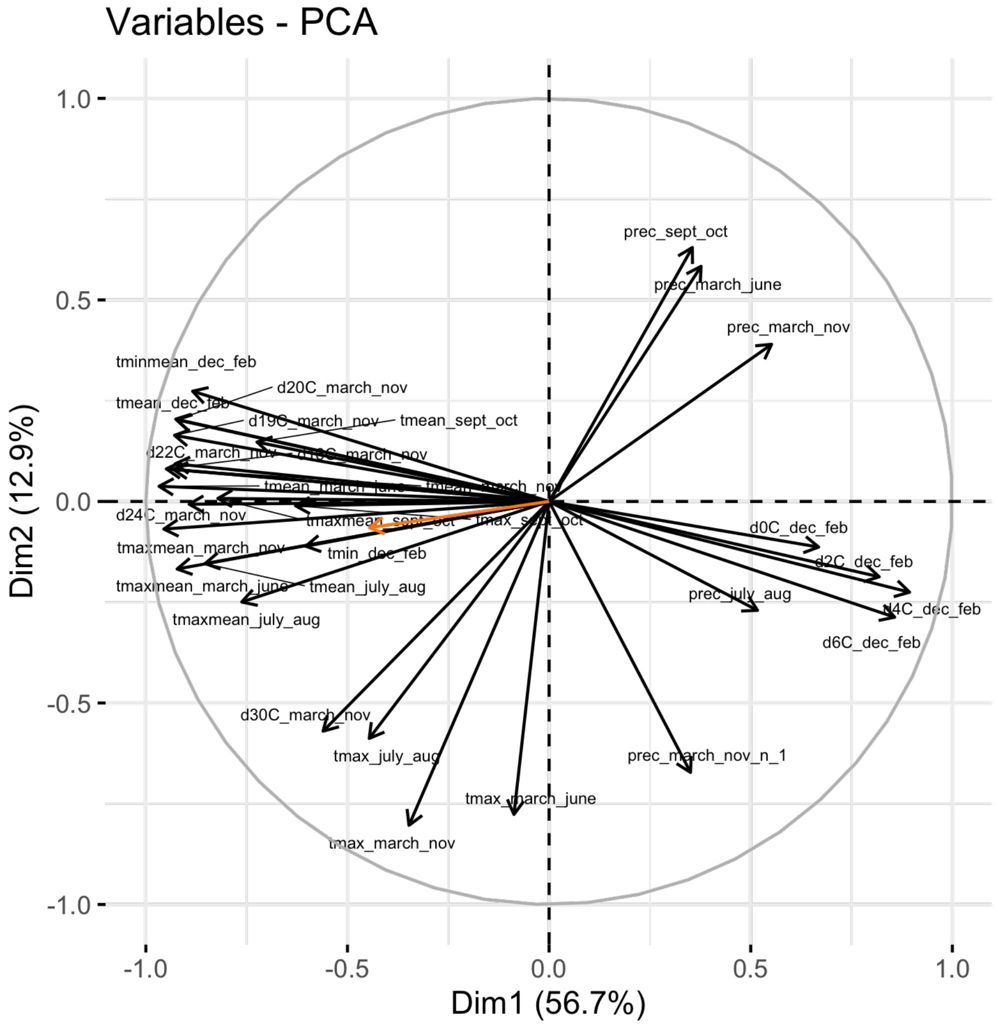

Figure S2 Spearman correlation matrix of climate variables calculated from SAFRAN data

Climate variables are available from Table S1a. Figure made with the R package corrplot [61].

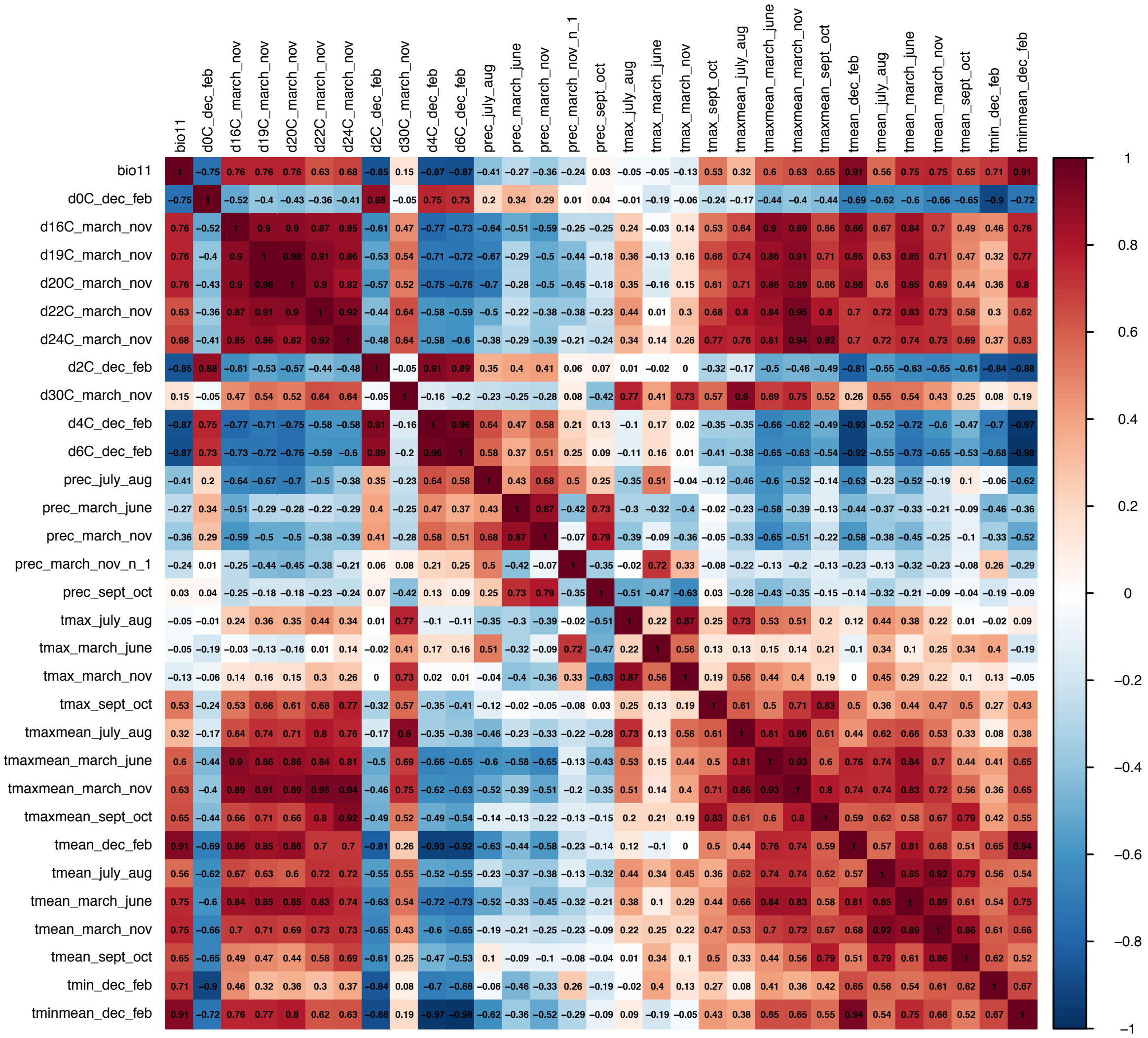

Figure S3 Climate suitability for *P. spumarius* (Ps) and *X. fastidiosa* (Xf) according to reference climate condition (1981-2010) and for the periods 2011-2040, 2041-2070 and 2071-2100 for the shared socio-economic pathway SSP126

Suitabilities for 1981-2010 are outputs of the model. Suitabilities for future periods are the median (consensus model) of the values obtained by projecting the model using the GCM accounted for in the study (see methods section for details).

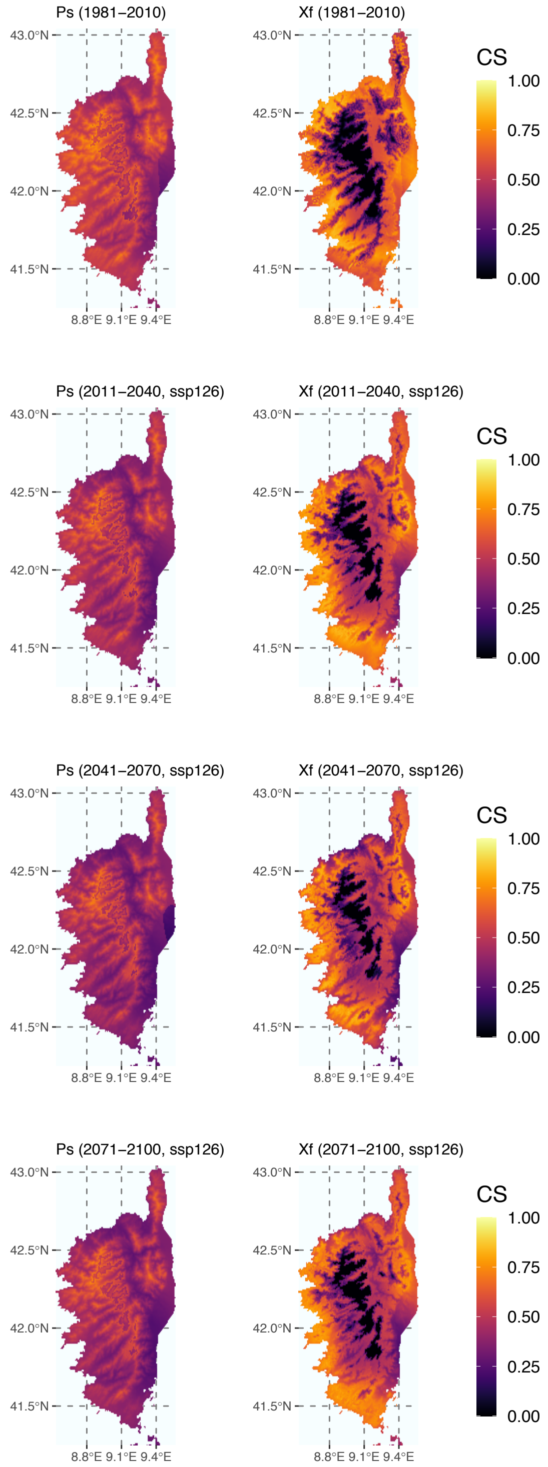

Figure S4 **Consensus models showing the of climate suitability for *P. spumarius* (Ps) and *X. fastidiosa* (Xf) for 2071-2100 for the shared socio-economic pathways SSP370 and SSP585**

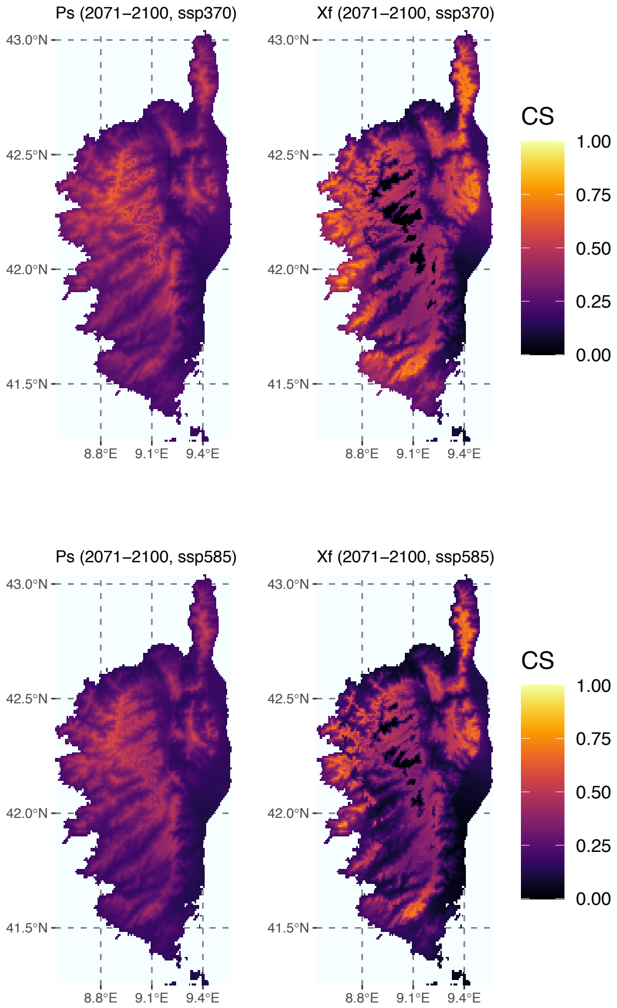

Figure S5 Scatterplots of raw data and GLMM predictions for pairwise associations between prevalence of *Xylella fastidiosa* in populations of vector and sampling year

A GLMM1. **B** GLMM2. Error bars on scatterplots: 95% confidence intervals. Letters: pairwise comparison of estimated marginal means; distributions sharing a letter do not differ significantly. Points: raw data jittered along abscissa.

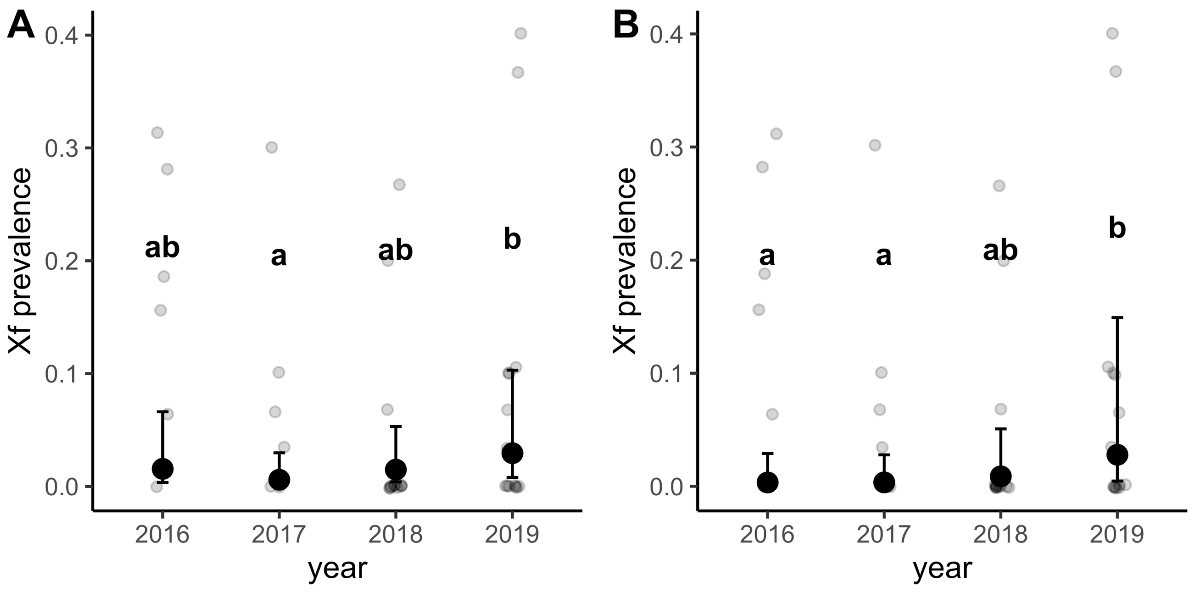

Cited references

1. EPPO PM 7/24 (4) *Xylella Fastidiosa*. *EPPO Bull.* **2019**, *49*, 175–227, doi:10.1111/epp.12575.
2. Cruaud, A.; Gonzalez, A.-A.; Godefroid, M.; Nidelet, S.; Streito, J.-C.; Thuillier, J.-M.; Rossi, J.-P.; Santoni, S.; Rasplus, J.-Y. Using Insects to Detect, Monitor and Predict the Distribution of *Xylella Fastidiosa*: A Case Study in Corsica. *Sci. Rep.* **2018**, *8*, doi:10.1038/s41598-018-33957-z.
3. Yuan, X.; Morano, L.; Bromley, R.; Spring-Pearson, S.; Stouthamer, R.; Nunney, L. Multilocus Sequence Typing of *Xylella Fastidiosa* Causing Pierce's Disease and Oleander Leaf Scorch in the United States. *Phytopathology* **2010**, *100*, 601-611.
4. Cruaud, P.; Rasplus, J.-Y.; Rodriguez, L.J.; Cruaud, A. High-Throughput Sequencing of Multiple Amplicons for Barcoding and Integrative Taxonomy. *Sci. Rep.* **2017**, *7*, 41948.
5. Marshall, O.J. PerlPrimer: Cross-Platform, Graphical Primer Design for Standard, Bisulphite and Real-Time PCR. *Bioinformatics* **2004**, *20*, 2471–2472, doi:10.1093/bioinformatics/bth254.
6. Marcelletti, S.; Scortichini, M. Genome-Wide Comparison and Taxonomic Relatedness of Multiple Xylella Fastidiosa Strains Reveal the Occurrence of Three Subspecies and a New Xylella Species. *Arch. Microbiol.* **2016**, *198*, 803–812, doi:10.1007/s00203-016-1245-1.
7. Martin, J.-F. Creating Error-Proof Indexes for High Throughput Sequencing 2019.
8. Bolger, A.M.; Lohse, M.; Usadel, B. Trimmomatic: A Flexible Trimmer for Illumina Sequence Data. *Bioinformatics* **2014**, *30*, 2114–2120.
9. Magoc, T.; Salzberg, S.L. FLASH: Fast Length Adjustment of Short Reads to Improve Genome Assemblies. *Bioinformatics* **2011**, *27*, 2957–2963, doi:10.1093/bioinformatics/btr507.
10. Mahe, F.; Rognes, T.; Quince, C.; de Vargas, C.; Dunthorn, M. Swarm v2: Highly-Scalable and High-Resolution Amplicon Clustering. *Peerj* **2015**, *3*, e1420, doi:10.7717/peerj.1420.
11. Rognes, T.; Flouri, T.; Nichols, B.; Quince, C.; Mahé, F. VSEARCH: A Versatile Open Source Tool for Metagenomics. *PeerJ* **2016**, *4*, e2584, doi:10.7717/peerj.2584.
12. Katoh, K.; Standley, D.M. MAFFT Multiple Sequence Alignment Software Version 7: Improvements in Performance and Usability. *Mol. Biol. Evol.* **2013**, *30*, 772–780.

13. Albre, J.; Carrasco, J.M.G.; Gibernau, M. Ecology of the Meadow Spittlebug *Philaenus Spumarius* in the Ajaccio Region (Corsica) – I: Spring. *Bull. Entomol. Res.* **2021**, *111*, 246–256, doi:10.1017/S0007485320000711.
14. Feil, H.; Purcell, A.H. Temperature-Dependent Growth and Survival of *Xylella Fastidiosa* in Vitro and in Potted Grapevines. *Plant Dis.* **2001**, *85*, 1230–1234, doi:10.1094/PDIS.2001.85.12.1230.
15. Feil, H.; Feil, W.S.; Purcell, A.H. Effects of Date of Inoculation on the Within-Plant Movement of *Xylella Fastidiosa* and Persistence of Pierce's Disease within Field Grapevines. *Phytopathology* **2003**, *93*, 244–251, doi:10.1094/PHYTO.2003.93.2.244.
16. Wistrom, C.; Purcell, A.H. The Fate of <i>Xylella Fastidiosa<\i> in Vineyard Weeds and Other Alternate Hosts in California. *Plant Dis.* **2005**, *89*, 994–999.
17. Lieth, J.H.; Meyer, M.M.; Yeo, K.-H.; Kirkpatrick, B.C. Modeling Cold Curing of Pierce's Disease in *Vitis Vinifera* 'Pinot Noir' and 'Cabernet Sauvignon' Grapevines in California. *Phytopathology*^{TM} **2011**, *101*, 1492–1500, doi:10.1094/PHYTO-08-10-0207.
18. Purcell, A. Paradigms: Examples from the Bacterium *Xylella Fastidiosa*. *Annu. Rev. Phytopathol.* **2013**, *51*, 339–356, doi:10.1146/annurev-phyto-082712-102325.
19. Daugherty, M.P.; Zeilinger, A.R.; Almeida, R.P. Conflicting Effects of Climate and Vector Behavior on the Spread of a Plant Pathogen. *Phytobiomes J.* **2017**, *1*, 46–53.
20. Daugherty, M.P.; Cooper, M.; Smith, R.; Varela, L.; Almeida, R.P.P. Has Climate Contributed to a Pierce's Disease Resurgence in North Coast Vineyards? *Pract. Winery Vineyard* **2019**.
21. Hewitt, W.B.; Frazier, N.W.; Freitag, J.H.; Winkler, A.J. Pierce's Disease Investigations. *Hilgardia* **1949**, *19*, 207–264.
22. Quintana-Seguí, P.; Le Moigne, P.; Durand, Y.; Martin, E.; Habets, F.; Baillon, M.; Canellas, C.; Franchisteguy, L.; Morel, S. Analysis of Near-Surface Atmospheric Variables: Validation of the SAFRAN Analysis over France. *J. Appl. Meteorol. Climatol.* **2008**, *47*, 92–107, doi:10.1175/2007JAMC1636.1.
23. R Core Team R Version 3.5.1 (Feather Spray): A Language and Environment for Statistical Computing. R Foundation for Statistical Computing, Vienna, Austria. URL Https://Www.R-Project.Org/. **2018**.
24. Brooks, M.E.; Kristensen, K.; van Benthem, K.J.; Magnusson, A.; Berg, C.W.; Nielsen, A.; Skaug, H.J.; Maechler, M.; Bolker, B.M. GlmmTMB Balances Speed and Flexibility among Packages for Zero-Inflated Generalized Linear Mixed Modeling. *R J.* **2017**, *9*, 378–400.

25. Chessel, D.; Dufour, A.-B.; Thioulouse, J. The Ade4 Package – I: One-Table Methods. *R News* **2004**, *4*, 5–10.
26. Wold, S.; Sjöström, M.; Eriksson, L. PLS-Regression: A Basic Tool of Chemometrics. *Chemom. Intell. Lab. Syst.* **2001**, *58*, 109–130, doi:10.1016/S0169-7439(01)00155-1.
27. Mehmood, T.; Liland, K.H.; Snipen, L.; Sæbø, S. A Review of Variable Selection Methods in Partial Least Squares Regression. *Chemom. Intell. Lab. Syst.* **2012**, *118*, 62–69, doi:10.1016/j.chemolab.2012.07.010.
28. Dormann, C.F.; Elith, J.; Bacher, S.; Buchmann, C.; Carl, G.; Carré, G.; Marquéz, J.R.G.; Gruber, B.; Lafourcade, B.; Leitão, P.J.; et al. Collinearity: A Review of Methods to Deal with It and a Simulation Study Evaluating Their Performance. *Ecography* **2013**, *36*, 27–46, doi:10.1111/j.1600-0587.2012.07348.x.
29. Faraway, J.J. *Extending the Linear Model with R: Generalized Linear, Mixed Effects and Nonparametric Regression Models*; Chapman and Hall/CRC: Boca Raton, FL, 2006;
30. Bolker, B.M.; Brooks, M.E.; Clark, C.J.; Geange, S.W.; Poulsen, J.R.; Stevens, M.H.H.; White, J.-S.S. Generalized Linear Mixed Models: A Practical Guide for Ecology and Evolution. *Trends Ecol. Evol.* **2009**, *24*, 127–135, doi:10.1016/j.tree.2008.10.008.
31. Hartig, F. *DHARMa: Residual Diagnostics for Hierarchical (Multi-Level / Mixed) Regression Models*; 2020;
32. Fox, J.; Weisberg, S. *An {R} Companion to Applied Regression*; Third Edition. Thousand Oaks.; 2019;
33. Lenth, R.V. *Emmeans: Estimated Marginal Means, Aka Least-Squares Means*; 2021;
34. Hothorn, T.; Bretz, F.; Westfall, P. Simultaneous Inference in General Parametric Models. *Biom. J.* **2008**, *50*, 346–363, doi:10.1002/bimj.200810425.
35. Karger, D.N.; Conrad, O.; Böhner, J.; Kawohl, T.; Kreft, H.; Soria-Auza, R.W.; Zimmermann, N.E.; Linder, H.P.; Kessler, M. Climatologies at High Resolution for the Earth's Land Surface Areas. *Sci. Data* **2017**, *4*, 170122, doi:10.1038/sdata.2017.122.
36. Xu, T.; Hutchinson, M. ANUCLIM Version 6.1 User Guide. *Aust. Natl. Univ. Fenner Sch. Environ. Soc. Canberra* **2011**.
37. Hijmans, R.J.; Phillips, S.; Leathwick, J.; Elith, J. *Dismo: Species Distribution Modeling*; 2017;
38. Karger, D.N.; Conrad, O.; Böhner, J.; Kawohl, T.; Kreft, H.; Soria-Auza, R.W.; Zimmermann, N.E.; Linder, H.P.; Kessler, M. Climatologies at High Resolution for the Earth's Land Surface Areas. *Sci. Data* **2017**, *4*, 170122, doi:10.1038/sdata.2017.122.

39. Karger, D.N.; Schmatz, D.R.; Dettling, G.; Zimmermann, N.E. High-Resolution Monthly Precipitation and Temperature Time Series from 2006 to 2100. *Sci. Data* **2020**, *7*, 248, doi:10.1038/s41597-020-00587-y.
40. Buisson, L.; Thuiller, W.; Casajus, N.; Lek, S.; Grenouillet, G. Uncertainty in Ensemble Forecasting of Species Distribution. *Glob. Change Biol.* **2010**, *16*, 1145–1157, doi:10.1111/j.1365-2486.2009.02000.x.
41. O'Neill, B.C.; Kriegler, E.; Riahi, K.; Ebi, K.L.; Hallegatte, S.; Carter, T.R.; Mathur, R.; van Vuuren, D.P. A New Scenario Framework for Climate Change Research: The Concept of Shared Socioeconomic Pathways. *Clim. Change* **2014**, *122*, 387–400, doi:10.1007/s10584-013-0905-2.
42. Godefroid, M.; Cruaud, A.; Streito, J.-C.; Rasplus, J.-Y.; Rossi, J.-P. *Xylella Fastidiosa*: Climate Suitability of European Continent. *Sci. Rep.* **2019**, *9*, 8844, doi:10.1038/s41598-019-45365-y.
43. Falsini, S.; Tani, C.; Sambuco, G.; Papini, A.; Faraoni, P.; Campigli, S.; Ghelardini, L.; Bleve, G.; Rizzo, D.; Ricciolini, M.; et al. Anatomical and Biochemical Studies of Spartium Junceum Infected by Xylella Fastidiosa Subsp. Multiplex ST 87. *Protoplasma* **2021**, doi:10.1007/s00709-021-01640-2.
44. Boria, R.A.; Olson, L.E.; Goodman, S.M.; Anderson, R.P. Spatial Filtering to Reduce Sampling Bias Can Improve the Performance of Ecological Niche Models. *Ecol. Model.* **2014**, *275*, 73–77, doi:10.1016/j.ecolmodel.2013.12.012.
45. Aiello-Lammens, M.E.; Boria, R.A.; Radosavljevic, A.; Vilela, B.; Anderson, R.P. SpThin: An R Package for Spatial Thinning of Species Occurrence Records for Use in Ecological Niche Models. *Ecography* **2015**, *38*, 541–545, doi:10.1111/ecog.01132.
46. Phillips, S.J.; Anderson, R.P.; Schapire, R.E. Maximum Entropy Modeling of Species Geographic Distributions. *Ecol. Model.* **2006**, *190*, 231–259, doi:10.1016/j.ecolmodel.2005.03.026.
47. Phillips, S.J.; Dudík, M.; Elith, J.; Graham, C.H.; Lehmann, A.; Leathwick, J.; Ferrier, S. Sample Selection Bias and Presence-Only Distribution Models: Implications for Background and Pseudo-Absence Data. *Ecol. Appl.* **2009**, *19*, 181–197.
48. Naimi, B.; Hamm, N.A.S.; Groen, T.A.; Skidmore, A.K.; Toxopeus, A.G. Where Is Positional Uncertainty a Problem for Species Distribution Modelling? *Ecography* **2014**, *37*, 191–203, doi:10.1111/j.1600-0587.2013.00205.x.

49. Varela, S.; Anderson, R.P.; García-Valdés, R.; Fernández-González, F. Environmental Filters Reduce the Effects of Sampling Bias and Improve Predictions of Ecological Niche Models. *Ecography* **2014**, 1084–1091, doi:10.1111/j.1600-0587.2013.00441.x.
50. VanDerWal, J.; Shoo, L.P.; Graham, C.; Williams, S.E. Selecting Pseudo-Absence Data for Presence-Only Distribution Modeling: How Far Should You Stray from What You Know? *Ecol. Model.* **2009**, *220*, 589–594, doi:10.1016/j.ecolmodel.2008.11.010.
51. Seabra, S.G.; Rodrigues, A.S.B.; Silva, S.E.; Neto, A.C.; Pina-Martins, F.; Marabuto, E.; Thompson, V.; Wilson, M.R.; Yurtsever, S.; Halkka, A.; et al. Population Structure, Adaptation and Divergence of the Meadow Spittlebug, *Philaenus Spumarius* (Hemiptera, Aphrophoridae), Revealed by Genomic and Morphological Data. *PeerJ* **2021**, *9*, e11425, doi:10.7717/peerj.11425.
52. Vollering, J.; Halvorsen, R.; Mazzoni, S. The MIAmaxent R Package: Variable Transformation and Model Selection for Species Distribution Models. *Ecol. Evol.* **2019**, *9*, 12051–12068, doi:10.1002/ece3.5654.
53. Mazzoni, S.; Halvorsen, R.; Bakkestuen, V. MIAT: Modular R-Wrappers for Flexible Implementation of MaxEnt Distribution Modelling. *Ecol. Inform.* **2015**, *30*, 215–221.
54. Fielding, A.H.; Bell, J.F. A Review of Methods for the Assessment of Prediction Errors in Conservation Presence/Absence Models. *Environ. Conserv.* **1997**, *24*, 38–49.
55. Hirzel, A.H.; Le Lay, G.; Helfer, V.; Randin, C.; Guisan, A. Evaluating the Ability of Habitat Suitability Models to Predict Species Presences. *Ecol. Model.* **2006**, *199*, 142–152, doi:10.1016/j.ecolmodel.2006.05.017.
56. Yackulic, C.B.; Chandler, R.; Zipkin, E.F.; Royle, J.A.; Nichols, J.D.; Campbell Grant, E.H.; Veran, S. Presence-Only Modelling Using MAXENT: When Can We Trust the Inferences? *Methods Ecol. Evol.* **2013**, *4*, 236–243, doi:10.1111/2041-210x.12004.
57. Streito, J.-C.; Chartois, M.; Pierre, É.; Dusoulier, F.; Armand, J.-M.; Gaudin, J.; Rossi, J.-P. Citizen Science and Niche Modeling to Track and Forecast the Expansion of the Brown Marmorated Stinkbug Halyomorpha Halys (Stål, 1855). *Sci. Rep.* **2021**, *11*, 11421, doi:10.1038/s41598-021-90378-1.
58. Broennimann, O.; Cola, V.D.; Guisan, A. *Ecospat: Spatial Ecology Miscellaneous Methods*; 2020;
59. Fadrosh, D.W.; Ma, B.; Gajer, P.; Sengamalay, N.; Ott, S.; Brotman, R.M.; Ravel, J. An Improved Dual-Indexing Approach for Multiplexed 16S RRNA Gene Sequencing on the Illumina MiSeq Platform. *Microbiome* **2014**, *2*, 1.

60. Kassambara, A.; Mundt, F. *Factoextra: Extract and Visualize the Results of Multivariate Data Analyses*; 2017;
61. Wei, T.; Simko, V. *R Package "Corrplot": Visualization of a Correlation Matrix*; 2017;
