# Supplementary material for: Vectors as Sentinels: Rising Temperatures Increase the Risk of Xylella fastidiosa Outbreaks
Source: Biology (Basel). 2022 Aug 31;11(9):1299. doi: 10.3390/biology11091299 (PMC9495951; doi:10.3390/biology11091299)
Supplement: Supplementary file 1 [file biology-11-01299-s001.zip › Supplementary_Map.html]

Supplementary map to


# Supplementary map to

### Vectors as Sentinels: Rising Temperatures Increase the Risk of *Xylella fastidiosa* Outbreaks

Pauline Farigoule, Marguerite Chartois, Xavier Mesmin, Maxime Lambert, Jean-Pierre Rossi, Jean-Yves Rasplus, and Astrid Cruaud

**Distribution of sampling sites and landscape vegetation structure within 1000 meters radius buffers zones around sites**

The vegetation structure was retrieved from the OCS GE database (© IGN – 2022, https://geoservices.ign.fr/ocsge). This database provided by the French National Institute of Geographic and Forestry Information includes land cover and land human use, photo-interpreted from 50cm-pixels orthophotographs, in the form of a polygon database which contours are positioned with a precision of ca. 1m.
